# Structural violence and the need for compassionate use of methadone in Mexico

**DOI:** 10.1186/s12889-022-12955-x

**Published:** 2022-03-29

**Authors:** Martha Romero-Mendoza, Ingris Peláez-Ballestas, Ariagor Manuel Almanza-Avendaño, Emilia Figueroa

**Affiliations:** 1 Instituto Nacional de Psiquiatría Ramón de La Fuente Muñiz, Camino a Xochimilco 101, Col, San Lorenzo Huipulco, CDMX, 144370 Tlalpan, Mexico City, Mexico; 2 Hospital General de México Dr. Eduardo Liceaga, Dr. Balmis 148, Col. Doctores, CDMX, 06720 Cuauhtémoc, Mexico City, Mexico; 3 Facultad de Ciencias Humanas, Universidad Autónoma de Baja California, Calz. Castellón S/N, Esperanza Conjunto Urbano, 21350 Mexicali, BC Mexico; 4 Clínica Integral de Tratamiento Contra Las Adicciones, Adolfo Prieto 1338, Col. Del Valle, Benito Juárez, CDMX, 03100 Mexico City, Mexico

**Keywords:** Structural violence, Methadone, Pilgrimage, Self-care, Treatment barriers, Mexico

## Abstract

**Background:**

Epidemiological data from Mexico have documented an increase in heroin use in the last decade. However, there is no comprehensive care strategy for heroin users, especially those who have been accused of a crime. The objective of this study was to describe the heroin and methadone use of intravenous heroin users of both sexes who have been in jail, to offer evidence for the formulation of health policy.

**Methods:**

This study used an ethnographic approach, with open-ended interviews carried out from 2014 to the present. Heroin users of both sexes attending a private methadone clinic in Mexico City were invited to participate. The sample was non-probabilistic. All interviews were audiotaped and transcribed, and narratives were analyzed using thematic analysis.

**Results:**

Participants in this study were 33 users of heroin, two of them women, who had been in prison. They ranged in age from 33 to 62 years, had used heroin for a period of 13–30 years, and were from three states: Michoacan, Oaxaca, and Mexico City. Three principal categories of analysis were structured: 1. Pilgrimage for help (dynamics of the drama of suffering, pain, and time through health care spaces); 2) methadone use as self-care; and 3) accessibility to methadone treatment. The impossibility of access to methadone treatment is a condition which motivates users in their journey. The dynamics of methadone use are interpreted as a form of self-care and care to avoid substance use. Reducing the psychological, physical, and harmful effects of the substance allows them to perform daily activities. The inability to access treatment leads to a significant effect on users who experience structural violence.

**Conclusion:**

Compassionate methadone treatment and holistic attention should be considered as a way to meet patients’ needs and mitigate their suffering, based on public health policy that allows for human rights-based care.

**Supplementary Information:**

The online version contains supplementary material available at 10.1186/s12889-022-12955-x.

## Background

Opiate use disorder has been defined as a biopsychosocial disorder where the likelihood of using drugs and their associated harms are influenced by genetic factors, early development, mental illness, social rules, exposure to the substance, and its market availability [1]. A large number of individuals who develop this disorder recover through a gradual or quick reduction in use, or the maintenance of a low level of use [2]. Others follow a course which may last for years or decades, with varying experiences of exposure, imprisonment, withdrawal, or death. It has been reported that one out of seven users do not respond to treatment [2]. In 2017 there were 47,506 opiate-related deaths in the United States (US) [3].

Medical treatment for opiate-related disorders includes various pharmacological agents: agonists of opiate µ-receptors such as methadone, partial agonists such as buprenorphine, antagonists such as naltrexone, and other approaches, such as lofexidine to handle withdrawal [1, 3]. This opioid substitution therapy is effective in reducing heroin use, human immunodeficiency virus (HIV) risk, and mortality. However, the treatment is often episodic, and it is difficult to maintain adherence, given that users also generally suffer from harassment and arrest, and these human rights violations interfere with their therapeutic trajectories and contribute to high morbidity and mortality [4, 5].

In the U.S., about one-third of heroin users enter correctional institutions every year. Only a few of these receive drug-assisted treatment (methadone or buprenorphine) for opiate use during imprisonment, and nearly three-fourths relapse into heroin use once they are released [6]. Involvement in criminal activities is greater among men, while women present a greater number of psychological problems [7].

In Mexico, research on heroin use has long focused on epidemiological studies and surveys of marginal populations on the country’s northern border [8–10]. However, use of the drug has expanded in the last ten years to other Mexican states, including Oaxaca, Michoacán, Nayarit, Puebla, and Chiapas [11, 12]. According to the United Nations Office on Drugs and Crime [13], there were 25,200 hectares of poppies cultivated in Mexico in 2015–16, with an increase of 5400 hectares (21%) in 2017, mostly in the northern states of Sinaloa, Chihuahua, Durango, and northern Nayarit, and in the southern states of Guerrero and Oaxaca, in the Sierra Madre del Sur. In of all these places there are drug cartels that generate social problems.

The production of illicit crops is practically the only way to survive in the poorest rural areas, such as Guerrero and Oaxaca. According to Álvarez-Rodríguez [14], opium poppies and marijuana, unlike corn, are commercial crops that provide an economic return in a region where money is scarce. Income from illicit crops is invested in home repair or the education of children. This production has created a variety of actors who have risen from laborers and caretakers of poppy plots to economic bosses who control the production of narcotics in a entire municipality. Poor farmers turn to the production of illicit crops to survive. The rise and consolidation of a drug trafficking network has been linked to the use of violence to control this space. In many cases, illicit crops are hidden in large fields to prevent detection by the military. It is when agreements are broken over who gives orders and who obeys that armed violence is used to impose or reestablish control over a territory, a population, or a crop [14].

In the state of Michoacán, this violence is linked to avocado production. According to Curry [15], Mexico is the world’s largest producer and exporter of avocados. In the last decade it has harvested an average of 1.56 million tons a year, 80% of which come from Michoacán. With the growth of this crop since the 1950s, the illegal planting of marijuana and opium has also grown. These crops have had a profound economic, social, and environmental impact [16]. Although the economic gains have been enormous, they have also been inequitable, and the population continues to suffer from impoverishment, stagnant wages, and a rising cost of living. There have also been problems of deforestation, water shortages, extorsion by organized crime, and health problems caused by the use of pesticides.

The state with the least information available concerning opium production is Oaxaca. Tamariz [17] notes that marijuana and opium provide the farmers of this state not only with a monetary income, but also with the possibility of continuing an agricultural life and avoiding the need to emigrate.

From 1990 to 2019, a total of 14,423 hectares of opium fields have been destroyed, and there have been 1857 murders committed in the two municipalities where most of these fields were located. Frissard, Farfán-Mendez, and Lecour Grandmaison [18] characterize this practice of destroying fields as a form of violence: it represents an economic loss of investment in labor and resources that affects only the farmers, not the intermediaries or the drug traffickers. The permanent presence of the military also reflects an arrangement of power that treats certain regions differently, and that contributes to the criminalization of poverty and the stigmatization of their inhabitants.

Among the undesirable results of the production, transportation, and sale of opium in these states is the ready availability of this substance to the population, and the beginning of opium and heroin use. To be a substance user in this context is not easy. It is not uncommon for substance users of both sexes to be stigmatized and forced to flee, while having to look for help to gain access to methadone. These dangerous environments have been defined by McGowan [19] as “social risk environments including discrimination and some other factors which bring about social disadvantages and physical risks (including violence and the harms inherent to impurities and adulterations in the drug supply)” (p. 1).

The objective of this study is to investigate the experiences of intravenous heroin users of both sexes who have been in prison, with attention to the pilgrimage they have undertaken in their search for help, self-care, and treatment. It seeks to provide evidence for policy-making in health care in areas where current circumstances favor internal displacement.

## Method

This study uses an ethnographic approach [20, 21] with semi-structured open-ended interviews [22] to give an account of the experience of heroin users of both sexes with use of this drug, and their eventual outpatient treatment in a private ambulatory methadone clinic in Mexico City. The study began in 2014 and is continuing in 2022. It was approved by the Research and Ethics Committees of the Instituto Nacional de Psiquiatría.

Prospective participants were introduced to the first author by the treating medical specialist or the person in charge of supplying the methadone. The physician evaluated whether prospective participants were free from the effects of heroin and thus able to take part in an interview. Appropriate candidates were provided with a written invitation. The research project was described to them also orally, in the presence of the physician and a clinic administrator. After a candidate agreed to participate, written or verbal informed consent was obtained from all participants and the interview was conducted in a private space.

In accordance with ethical principles, interviews were voluntary and confidential. Participants were asked permission to make an audio recording of the interview, and they were asked to choose a pseudonym. They were informed that the interview would be used exclusively for scientific purposes, and that they could change their mind at any time and withdraw their consent. They were then asked to sign the informed consent document in front of two witnesses that also signed. Some candidates decided to participate and agreed to the audio recording but not to signing the informed consent, saying that in the past they had been deceived into signing other types of documents. In these cases, an audio recording was made of their informed consent and two witnesses also signed the informed consent form, according to the legislation that states, that in minimum risk research projects, as the present one: “the formalization of obtaining consent can have several alternatives: express it clearly verbally or sign a consent form. Verbal acceptance has the characteristic of leaving it up to researcher to provide proof or evidence of acceptance” (p. 3) [23].

This formal introduction process served as an icebreaker. Male participants often joked about the pseudonyms they would use, choosing celebrities’ names and running to the restroom to comb their hair and tidy themselves up. One female participant sometimes agreed to be interviewed, but would then reschedule appointment for another day, fail to show up, and ask for another opportunity. One finally appeared at the third appointment, saying: “I wanted to test how much you were really interested in listening to me.” The interviewer always took care not to pressure participants**.**

### Data collection and sampling

From 2014 to the present (January 2022), 110 patients have been approached, of whom ten declined to participate, most of them women. Ten others did not return to the clinic. The participants in the final sample had spent time in prison and received treatment at the clinic in the period from 2014 to 2018.

The semi-structured open-ended interviews were the product of a social interaction that took place in a context of strict time limits and unequal status [22]. The duration of the interview was determined by each participant: they lasted from 20 min to two hours. The following thematic questions were asked of each participant: “How did you start using heroin?” and “How did you end up in this clinic?”, and some special cues were used to elucidate the narrative This interview guide (Supplementary file 1) was developed specifically for the study. If the participant wanted to share additional information, they were given the opportunity, and if they wished, they could return for further interviewing.

The clinic established some requirements: there could be only one interviewer, no contact was permitted outside the clinic between participant and interviewer, and there could be no exchange of telephone information. If the participant wanted to talk more about their experience, they had to make an appointment with clinic staff. If a participant needed information or psychiatric assistance due to the severity of their symptoms, they were referred to appropriate medical providers. No private information could be consulted from their medical records. The clinic has security cameras installed and the researcher consented to video surveillance.

As a methodological resource, these interviews were selective and intermittent, allowing at times for brief but continuous exchanges or longer periods of observation of the participants’ body language “construed as a plot of meanings” [24]. In addition, their attire and language, their relationships with other heroin users and with the people accompanying them and with clinic personnel, and the social processes underlying their drug problems were all observed as well.

### Sampling

The participants were a non-probabilistic convenience sample, a recruitment technique that does not give the same selection opportunities to all members of the population. This technique means that there is no frame of reference for the sampling and the limits of the population are unknown. In this sample, the confidentiality of the identities and data of the subjects is a concern, because they may belong to stigmatized groups or may have been involved in illegal activity [25].

The selection criteria for the sample were having used heroin and having been imprisoned. The sample included 33 diverse participants. A first group included people from Oaxaca, where a methadone clinic was closed after three persons were killed on the premises, one of them the female treating physician. This group included two subgroups. The first was made up of small farmers, artisans, students, and informal workers (Tables 1 and 2). The average age in this subgroup was 33 years, with an average of 16 years using heroin. Only two participants had shorter histories of using the drug: five and eight years. The second subgroup (Table 3), consisting of two foreigners of a medium–high socioeconomic level living at neighboring beaches, were the oldest users in the study, with an average of 30 years using heroin. Participants from Mexico City formed a second group; in addition to being heroin users, some were also drug dealers (Table 4). Their average age was 36 years with an average of 13 years of use. Informal workers and male family heads from the state of Michoacán made up a third group, with an average age of 37 years and an average of 14 years of use (Table 5). Finally, a fourth group included two female participants who had been in prison: one 44 years old and another 28 years old (Table 6). Both had children, used heroin during their pregnancies, and their children had severe development and health problems. For this reason, the children were in the custody of close relatives. These women were at a greater social disadvantage: they had resorted to sex work to obtain economic resources, they had no access to reproductive health services, and they had been economically dependent on violent male users for long periods of time.

**Table 1:** Oaxaca

Pseudonym	Sex	Heroin type	Crime	Sentence	Heroin availability inside the prison	Access to methadone treatment in prison
Ángel	M	Black, Speedball	Robbery	A year and a half	Yes	No
Oleodegario	M	Black	Auto parts theft	Few days	Yes	No
Luis	M	Black, Speedball	Robbery	8 years	Yes	No
Francisco Gabriel	M	Speedball	Robbery	NA	Yes	No
Juan Pérez	M	Black	Robbery, possession of heroin	NA	Yes	No
Mario López	M	Black	Robbery, carrying a firearm	2 years, and 8 months	Yes	No
Pasgar	M	White	Robbery, injury	4 years	Yes	No
Jose Luis	M	black	Robbery	1 year	Yes	No
Miguel	M	Black	Robbery	NA	Yes	No
Lalo Conteras	M	Black, Speedball	Robbery	NA	Yes	No
Alberto Torres	M	Black	Auto parts theft	3 days	Yes	No

^*^NA Not Available-Not answer-The user does not recall it

**Table 2:** Oaxaca

Pseudonym	Sex	Heroin type	Crime	Sentence	Heroin availability inside the prison	Access to methadone treatment in prison
Luis Rojas	M	Black, White, Speedball	Heroin possession	15–20 days	Yes	No
Rubén	M	Black	Robbery	3 years and 3 months	Yes	No
Héctor	M	Speedball	NA	NA	Yes	No
Emmanuel	M	Black	Auto parts theft	NA	Yes	No
Brad Pitt	M	Black, White	Assault, carrying a firearm, drugs	2 years in penitentiary and 3 years in prison	Yes	No
John Smith	M	White	Extortion, organized crime	1 year and 7 months	Yes	No
Jose Luis 2	M	Speedball	Assault, carrying a firearm, drugs	10 years	Yes	No
Vitor	M	Speedball	Heroin possession, organized crime	3 years	Yes	No
Jose Alfredo	M	Black	Assault, Robbery	4 years and 3 months	Yes	No
Alex lora	M	Black	Robbery, assault	3 months	Yes	No

^*^NA Not Available-Not answer-The user does not recall it

**Table 3:** Foreigners in oaxaca

Pseudonym	Sex	Heroin type	Crime	Sentence	Heroin availability inside the prison	Access to methadone treatment in prison
Enriqu	M	Black, White	Caught injecting heroin	3 months	Yes	No
El Chapo	M	Black, White, brown	Heroin possession	1 year	No	Yes

^*^NA Not Available-Not answer-The user does not recall it

**Table 4:** Mexico city

Pseudonym	Sex	Heroin type	Crime	Sentence	Heroin availability inside the prison	Access to methadone treatment in prison
Víctor (DEAD)	M	Black, White	Marijuana possession	NA	Yes	NA
Emmanuel	M	Black, Speedball	Assault, carrying a firearm, drugs	NA	Yes	No
Francisco	M	Black	Robbery	NA	Yes	No
Héctor Garibaldi	M	Black	Assault, carrying a firearm, drugs	NA	Yes	No

^*^NA Not Available-Not answer-The user does not recall it

**Table 5:** Michoacán

Pseudonym	Sex	Heroin type	Crime	Sentence	Heroin availability inside the prison	Access to methadone treatment in prison
Lucas	M	White, Black, blue	Robbery	8 months	Yes	No
Michael Douglas (DEAD)	M	Black	Robbery, homicide	5 years	Yes	No
Rogelio Manuel	M	Black, Speedball	Assault, Robbery	9 months	Yes	No
Jorge Eduardo	M	White, Black, Speedball	Drug possession	10 years and 6 months	Yes	No
Gerardo	M	Black, Speedball	NA	NA	Yes	No

^*^NA Not Available-Not answer-The user does not recall it

**Table 6:** Women

Pseudonym	Sex	Heroin type	Crime	Sentence	Heroin availability inside the prison	Access to methadone treatment in prison
Ámbar	F	White, Black	Assault, carrying a firearm, drugs, organized crime	NA	Yes	No
Fátima	F	Black	Sale of drugs, crimes against health	4 years	Yes	No

^*^NA Not Available-Not answer-The user does not recall it

Most users had used black tar heroin (42.42%), black and white heroin (21.21%); some had used “speedball,” a combination of cocaine with heroin (12,12%), black and speedball (21.21%) and only white (3.03%). In addition to injection drugs, users from Oaxaca frequently chew opium gum. In Mexican prisons, users might get heroin, but not methadone. Foreigners had been able to obtain methadone treatment in their country of origin.

This study has been ongoing for several years for two reasons. First, restrictions imposed by the Federal Commission on Health Risks (Comisión Federal para Riesgos Sanitarios, COFEPRIS) on methadone meant that there were four periods, one longer than eight months, when no methadone could be dispensed. Either its importation from the United States was prohibited, or there were administrative delays, or there was a transition to dispensing methadone produced in Mexico. Second, there were fluctuations in the number of users appearing at the clinic because of pressures from organized crime, which sometimes prevents them from going to the clinic and sometimes encourages it, given their need to travel to Mexico City in order to survive.

At various times, consideration was given to ending the study, but heroin users kept arriving from other locations. There was first an influx of heroin users from Oaxaca, followed by users from Mexico City. After a time, these stopped coming to the clinic, and others began to arrive from Michoacán. Lately, injection drug users have been arriving from other states. The data collection process will end when 100 participants have been interviewed.

The participants interviewed for the study are not foreign immigrants, they do not usually live in the street, they have not broken ties with their families, and most have a job, even if only a temporary one.

### Thematic analysis

All of the interviews were recorded, transcribed, and systematically analyzed. The approach used to review participants’ stories was narrative analysis, which Riessman [26] defines as “the family of methods [used] to interpret texts that share a historicized form” (p. 11). In this method, information retrieved from narratives is interpreted through the stories the individuals need to report, the chronology of successive events, and breaking points or epiphanies. A thematic analysis was also carried out, analyzing what was said or written during the collection of the information [27]. The analysis was carried out by the research team.

### Analysis

Data analysis followed the phases in Braun [28] and Clarke [29]: 1) familiarization with the data; 2) generation of initial codes and categories (coding features of the data in a systematic way across the entire dataset); 3) search for themes (gathering data relevant to each potential theme); 4) review of themes; 5) definition and naming of themes; and 6) writing.

The data was analyzed thematically using a mixture of deductive and inductive approaches. Reflexivity was improved with the involvement of the group in the analytical process, which ensured discussion and agreement on the themes generated [30]. The analyses were performed independently by two medical anthropologists and one psychologist and were reviewed for convergence of the themes identified; the thematic analysis was subsequently reviewed to reach agreement regarding the interpretation needed for data triangulation.

## Results

Three principal themes were structured: 1) pilgrimage for help; 2) methadone use as self-care; and 3) access to methadone treatment.

### Pilgrimage for Help

In Mexico, substance users diagnosed with dependence disorders have reported ten-year delays in starting treatment. Among the factors associated with these delays are impaired functioning, stigma associated with use, and availability of appropriate treatments [31]. The inability to access methadone treatment is a condition that motivates the user in their journey. This concept of a pilgrimage describes the path followed by the user and their family until they find relief for their biosociocultural distress. They undertake this journey through various health care spaces, both institutional and otherwise. The concept of pilgrimage also refers to a trajectory of pain, beginning with users and their families’ identification of symptoms as a problem. It also implies the search for explanations and solutions, taking into account the multiplicity of circumstances and their possible aggravating factors. The pilgrimage is part of the dynamics of the drama of suffering, and so it bears a relationship with pain, with space, and with time [32, 33]. The prolongation of the search contributes to the chronicity of substance use and the psychosocial deterioration of the user.

One of the main care services frequented by users are the internment centers commonly known as *anexos* (residential mutual aid centers). The *anexos* rarely provide methadone and treatment is focused on the psychological factors related to substance use, including moralistic interpretations. One problem in the *anexos* is that users suffer physical and emotional abuse, in violation of their human rights [34].

*“Nine bastards come for me, they get me up to wash, they tie me up, they wrap me in a blanket, and they take me. You know? So then they tie me up, shave my head, a cold-water bath. Some paddling, some slapping, and they had me standing for eight days in flip-flops…. Some, like those paddles for ice cream, this big, wrapped in duct tape.… Then they wet your butt, bend you over a desk, and “Put your ass in the air, motherfucker.” You know? I mean, it goes on: one, two, three whacks. You know?* On *the legs, the butt, wherever. It’s like getting hit with a paddle, you know, only bigger of course… And of course I’m all wet.... So, I mean, I can tell you, now, with something like satisfaction, you know? At the time, no, it was very, very hard. Like I was angry at God…. I felt angry with my family, because, well, it was terrible”. (Héctor, Mexico City)*

Another criticism users have of the *anexos* is that they create a kind of revolving door because relapses are frequent once patients leave, and the *anexos* offer help to families when substance users get out of control. However, they question the quality of the services provided as well as the cost of the treatment.

*“There were times when it was my parents who sent me because I stole from them. I took money from them, things, and they understood my addiction and they told me, we know you are not like that, we are going to support you, go to a rehab center, and I said ok, let’s go, and they took me. And I would stay there for two or three months, and then I would come out and start all over again”. (Lucas, Michoacán)*

Some users say they prefer to avoid the *anexos*, not only because they are not effective, but also because they think they might establish bonds with other users. This could imply a public acknowledgement of their substance use and cause a breach in their family relationships if their recovery is not successful.

*“And they come out, since they have lived through hell, they start doing drugs once again. Their family sees that they are using drugs again, and they try to help them again and they send them back. They get out, and even though they know their family is helping them, they don’t care. The family starts telling them, we’ve helped you once, twice, and still you don’t get it, and then they just let you do what you want”. (Hugo, Oaxaca)*

To a lesser degree, users also try psychosocial treatments that focus on change through words or conversation, the search for corrective emotional experiences, the management of behaviors associated with substance use, or the creation of social networks to reduce it. Users may receive these kinds of services through private consultation, telephone hotlines, self-help groups, and even when they are sent to prison.

*“The last time I was in prison, I gave it up for a year, a year and a half. I got into a group in prison; I was in a group called New Dawn… and it worked for me. It was talks, Christian talks most of all, and exercise, talks, distractions, not to keep doing the same in the cells because it’s different, there it was just working and using, working and using, there wasn’t anything else. [In the group] no, there were talks, exercise, and keeping yourself busy. When I was there something that helped me a lot was playing soccer, because there comes a time when they ask you if you want to stay with the group or you want to leave.” (José Alfredo, Oaxaca)*

Substance users may also receive outpatient psychiatric care or be admitted to psychiatric hospitals, usually when substance use has intensified, when they show symptoms that alarm them or their families, or when they have an emergency or crisis. Here they usually receive antipsychotic, anxiolytic, or antidepressant medications, but no specific treatment for heroin use, such as methadone.

*“He was seeing a psychiatrist from social security [health services]. First outside, the first time everything was private, a psychiatrist had treated him about a year, a private psychiatrist.… Then we went to social security. This same psychiatrist referred us there. He told us, if you want, take him to social security. At that time the psychiatrist’s appointments cost us about 350 pesos. He started charging us that, and with the medication, every session amounted to 1000 or 1500 pesos…. I remember the social security medications: olanzapine, clonazepam, and sertraline”. (Wife of Gerardo, Michoacán)*

In addition to the lack of methadone, heroin users need to face three other difficulties: the lack of specificity in treatment in terms of the substances used, the lack of a network to refer users to the right services, and a lack of comprehensive treatment that focuses both on the psychosocial and the biological dimensions of dependence. The concept of the pilgrimage seems to be favored in contexts where there are multiple ideologies about the treatment for substance dependence [35] and there is not enough regulation of treatment based on its effectiveness.

In such circumstances of therapeutic abandonment, it is common for users to try dealing with heroin use on their own, through sheer willpower or even with self-medication. However, lack of access to methadone will limit these individual efforts at recovery, and just as with care centers, there is a risk that substance use will eventually become chronic.

*“I haven’t gone [to treatment centers], I have tried to give it up on my own…. But you’re so stupid. I’d say to myself, I’ll try to reduce the dose one day, two days, and yes, I’m going to make it, but then on the third day I relapse and I smoke in one day what I didn’t in three”. (Hugo, Oaxaca)*

It has been found that substance users self-medicate because of the admission policies and limited access to opiate substitution treatments, difficulties in complying with treatment rules, and stigmatization, or when they find treatment controlling or authoritarian, or they lose their autonomy [36].

### Methadone use as self-care

Self-care refers to self-knowledge in a space of freedom [37]. It is the human relation with oneself and with one’s body, in the concrete sociocultural context where it is construed. Care is a continuum which encompasses self-care, care for others, and care for the social group where subjects coexist [38]. In the participants’ narratives, methadone use is interpreted as a form of self-care and care for the other, insofar it allows the substance user to look after their own body and also their family by avoiding substance use.

In the following fragments from the participants’ narratives, the self-care used in the personal, familial, and work environments produces a feeling of tranquility, of a return to “normalcy,” of avoiding the “existential vacuum” they experience and fill by using different drugs. The men and women interviewed are well acquainted with the various ways in which methadone renders them benefits, be they adaptive, psychological, or physical. They also recognize the adverse effects.

### Adaptive effects

Participants talked about the possibility of resuming their activities and reducing the problems they have with their families, while at the same time working without pain. One consequence, which may well be the most important, is that methadone keeps them away from criminal networks. An early study with health personnel identified some benefits of methadone substitution treatment, such as the ability to adopt a normal lifestyle and avoid criminal behavior [39].

*“In my opinion I think there is a need for more clinics such as this, because in Oaxaca there aren’t any. There used to be one but now it’s closed, and there is no denying it’s the only treatment that helps you feel well. I say this because I’ve taken several things, I have been taken to doctors, and it doesn’t help at all, I feel the same. [Methadone] is the only treatment I feel good with, the one with which I can live a peaceful, normal life. I can work without pain”. (Enrique, Oaxaca)*

*“Yes, it has served me really well, because when I don’t use heroin and I use methadone instead, I feel as if I was another person. I don’t do bad things like stealing from people, selling things I take from home, so many bad things, and the truth is I now regret it because I’ve gone to the extreme of working with people who used to sell”. (Luis, Oaxaca).*

*“For me, [methadone] is a miracle.... At least most people are not going to be on the street, stealing. As a society, no matter what your morality, it’s better not to have people stealing”. (Enrique, Oaxaca)*

*“What methadone does is, it doesn’t drug you, it just gets rid of feeling sick. With it, I can work normally, without worrying. You can eat, go out and work without worrying, without that desperate need to get something to get high”. (Juan Pérez, Oaxaca)*

### Psychological effects

The most valued effect of methadone is that it reduces anxiety and craving, which offers a sense of calmness that allows people to perform daily activities. The reduction of craving is a reason given by substance users to explain their preference for opiate substitution treatment [40].

*“The difference is that with methadone, I have, it has made me, it is a heroin substitute that calms anxiety, despair, and the sick feeling you have when you need heroin. I mean, methadone medication really works for me”. (José, Oaxaca)*

*Yes, it does take away the sick feeling. I mean, there’s no problem. Anxiety is the main thing, understand? The shakes—it does away with all that, and I guess to a certain extent it’s a drug, but it can be controlled. Why? Because the effect lasts longer and you can carry on doing things without using and thinking about the next dose, understand? (Luis Rojas, Oaxaca)*

*“I started coming and saw that the medication was working for me, and I started taking two pills a day, then one, and I spread it out like that, then half a pill, and when I’m not doing anything just a fourth of a pill. What I do is a lot of sports: I like boxing, swimming, playing soccer, and running. So, I stay active, and more than anything, I have work, I have a job, because that’s what I’ve seen, that it’s when I don’t do anything that I’m just looking to get into trouble.” (John Smith, Oaxaca)*

For the women, the obsession to use substances is reduced or even disappears, something which they translate into a reduction of suffering.

*“Having shifted to methadone has been very good for me because it blocks that obsessive side. Other treatments were a kind of suffering for me. To be honest, it was agony for me to go for a day, even a few hours, without using”. (Ámbar, Michoacán)*

*“I feel so much better; I can have a normal, normal, normal day. I can work, I eat well, I stop thinking about how to get money because the effect of the heroin will go away soon and I’ll feel bad, really bad, and so I’ll have to go get more. With methadone you stop thinking about all that: you’re calm. When you use heroin, the only thing you think about is that while you’re trying to get a hundred pesos, you’re going to feel really bad, because the effect of the dose you’ve just taken will soon be gone, and so you have to get another hundred pesos to go buy more when you start feeling really bad once again. It’s a never-ending story.... “(Fátima, Oaxaca)*

### Physical effects

The physical effects most frequently mentioned of heroin withdrawal were bone pain and diarrhea. Methadone is effective in eliminating the withdrawal syndrome. When it is not available, family members, usually their wives, bathe them in ice water to ease the pain.

*“I believe that methadone does work, I mean, it’s a matter of someone wanting to move on, and it also eases the suffering heroin causes. For the effects like the shakes, bone pain, diarrhea, sneezing, yawning, all of the effects, it may be that a lot of people say that it isn’t that bad, that’s it’s like a fever, but the fact is that the effects are very strong”. (Rubén, Oaxaca)*

### Harmful effects

Most participants said that methadone withdrawal is stronger than that of heroin. Withdrawal from methadone may lead to death, as in the case of Michael Douglas, who traveled to Mexico City in search of treatment, even though he already had respiratory problems. Methadone was prohibited at the time, and he died upon returning home. People cut off from methadone treatment may relapse to heroin after months or even years of being under control.

*“Methadone is like a [shot], it’s a fix. A fix is a problem. But it’s not a good fix. But they also used different formulas [naloxone] to treat people for heroin addiction; they used it like methadone. They take it every day, they put a pill on your tongue and let it go every day. They don’t give you any to take home, right? People don’t abuse it because if you take one it doesn’t do anything, that’s a fact; one pill a day and you feel strangely normal and that’s why I like it.” (El Chapo, Oaxaca)*

Some heroin users have reported fear at experiencing symptoms of withdrawal and of developing a methadone dependence [40]. Fear of addiction influences their preference for managing their heroin consumption or overcoming their addiction through willpower over methadone treatment [41].

“Unfortunately, sometimes you prefer to use heroin, because between heroin and methadone, the effects of [withdrawing from] methadone are worse”. (Héctor. Oaxaca)

### Access to methadone treatment

The principle of treatment accessibility is that professional, quality health services should be accessible to everyone. These services should be respectful of medical ethics, culturally appropriate, and sensitive to people’s needs, regardless of age or gender [42].

Lack of access to treatment has a major effect on heroin users who experience the dual vulnerability of being substance users and being in prison. In order to undertake methadone treatment, they have to resort to unorthodox strategies, such as taking advantage of prison authorities’ corruption or by smuggling it in with visiting family members.

*“They didn’t allow [methadone] in jail. They were doing it when there was a female doctor at the clinic. She eased the guys’ suffering by introducing it little by little. But the deal between the prison director and the prisoners’ leaders, there are connections, and that is how [heroin] gets in. They get it in the prison, and they themselves sell it. Every cell has some. (Mario, Oaxaca)*

They women in this group refer in particular to a lack of attention toward women seeking methadone treatment.

*“[Female substance users] do not know how to get to the clinic but they “get lost" with PVC [an inhalant], a drug, a yellow can, which is used to unblock drains. They soak a rag in it and they inhale it through their mouth. They soak the rag in that liquid, and they behave worse than if they were drunk. That’s why they don’t come anymore”. (Fátima, Oaxaca)*

Heroin users in prison are in a situation of greater structural vulnerability; they occupy a lower hierarchical position because of social inequalities like racism, sexism, classism, and the structural inequalities of poverty and criminalized drug use [43]. These inequalities limit their ability to look after themselves and increase their vulnerability in the face of biopsychosocial deterioration.

### Lack of knowledge of health professionals and authorities

Substance users perceive a limited technical competence on the part of health professionals, which constitutes a limitation to their access to health care. Medication-assisted treatment for substance dependence is limited, given the persistence of stigmatization of addiction and beliefs about change based on will power, in which evidence-based practices are sometimes considered ineffective [44]. Apart from integrating this type of treatment into primary care, it is necessary to develop the abilities of health care personnel to properly screen, treat, and refer patients who are substance users [39].

*“Another doctor used to tell me, [methadone] is [also] a drug, and I would tell him, yeah, but there is no comparing a medication which is controlled, and which they give to you, slowly reducing the dosage, and a drug you can go out and buy right now” (Oleodegario, Oaxaca)*

*“There were times when I had to get a special doctor so that I could buy the ordinary commercial bottle. And I would also ask him to spread the word about the kind of alternative methadone represents, just like they do in the United States, because there are many people who know about it there. Unfortunately, in Mexico, not even the authorities know what methadone is. There are authorities who have arrested us and they don’t even know what it is”. (Héctor, Oaxaca)*

### Health care in contexts of violence

Quality health care services offered by health care professionals may be limited by the violent context where care is provided. Interviewees mentioned a violent incident involving health care personnel at the methadone clinic in Oaxaca, which led to the closure of the clinic and a frantic search for methadone in other states, methadone smuggling, and the need to travel to Mexico City, which increased the cost of the treatment.

*“I was arrested in November, the doctor was killed in December, and all of us addicts had no clinic anymore, because even though we used methadone, heroin was sold out of the clinic or close by. We would buy methadone and we would go out to buy heroin anyway, and so it was useless. When I was already in jail, we all learned that the doctor had been killed”. (Fátima, Oaxaca)*

*“She [the doctor] was very nice, I liked her, she was a very nice woman. One day I went there and the police were all over the place. They were killing everybody. What a horrible waste! They had just killed everybody…”. (El Chapo, Oaxaca)*

### Private care for methadone treatment

Heroin users who want methadone treatment must go to private clinics: there are no public clinics that provide it. For a time, methadone treatment was subsidized by the Mexico City government, but a new administration canceled that program. The treatment is more costly in the beginning when it requires higher doses, and the cost has an effect on the user and their family.

*“When we come for a supply of the medication, whenever I come, I get 10 or 15 doses. Right now, I’m getting only five doses because I have no money. I’m telling you the truth: that’s all the money we have. There are times when my family helps me, and I get more, but right now they have to work and they are in need. So, as I was saying, we have to steal to get more doses. Right now, I have exactly enough for my five doses. These doses last only five or six days, and I’ll come back as soon as I get some more money, because this is an expense, but with this, thank God, we are no longer using”. (Víttor and Brad Pitt, Oaxaca).*

*“In my case I come from Oaxaca, and yes, it’s very expensive. In fact, I paid for my daily dose and it wasn’t hard for me, but the problem is you have to save enough money to come to [Mexico City], pay for methadone, the trip, food, and all that, and so it’s hard. If the clinic was [in Oaxaca], you pay daily, it’s not hard, with a job”. (Juan Pérez, Oaxaca)*

Even for heroin users who have access to services where they live, there are barriers to treatment, such as the expense of traveling to a clinic to get the dose and the cost of the medication [41]. These conditions particularly exclude structurally vulnerable people who lack economic resources, live in unstable households, or suffer criminalization for being substance users [43].

“There are times when you don’t have any money; there are times when there is no medication, and so what can you do?”. (Víttor and Brad Pitt, Oaxaca)

### Strategies for coping with methadone unavailability

Despite the unavailability or lack of access to methadone, users desperately seek alternative strategies, such as traveling to different parts of the country, searching online, and even buying methadone on the black market, with all of the underlying risks, to avoid relapsing into heroin use.

*“In fact, I was on the verge of quitting methadone; I was taking only twenty grams every other day. But then there were problems and it was cut off. And then, until I was able to go online and find out about the clinic in Mexico City, then we came back. But, as I was saying, there are times when they suspend it for months and you relapse. Well, at least you have the option to look somewhere else, you look elsewhere. [On the black market] the problem is that the strength is different. I don’t know if they cut it a lot; I wonder if it is methadone at all”. (Héctor, Oaxaca)*

“And in those two weeks when I didn’t have any methadone, I used heroin and I believe I used more in those 15 days than I did in a month before”. (Oleodegario, Oaxaca)

*“There’s a way to get methadone, but you need a prescription from a physician and it has to have a psychotropic medication stamp. You need this to get a bottle with a hundred 10 mg pills. I bought one once, but I wasn’t able to get another prescription”. (Alex Lora, Oaxaca)*

McNeil [43] notes that heroin users with structural vulnerabilities relapse because of withdrawal symptoms, or they buy methadone illegally. They may also resort to sex work or selling drugs to survive. In Mexico, this vulnerability is increased by the lack of local health care services and the obstacles to treatment in public clinics, which increase the cost of medication and the expense of getting to appointments.

*“I had a few [methadone] pills, for a week… but I went for a month without using any [heroin], and then there came the moment finally when I didn’t have enough to alleviate the symptoms and go on living. I couldn’t find it in Oaxaca. And then, finally, after a lot of research, I looked on the internet and I contacted a clinic in Mexicali, or Tijuana, I don’t remember which, and they sent me a list of all the clinics in Mexico.*

*So, I started coming here [Mexico City]. There was a problem with the paperwork and there was no medication; weeks and months went by. And at each one of those times, you have to do something. Even though I lowered my dose, I haven’t lowered it as much as I could.... So, you start all over again, using as much methadone as before, and it takes a few weeks, but you’re actually stable again…. I even looked for a place to get myself admitted for two weeks or something like that…. I didn’t have enough money, on the one hand, and really, where would they take care of me? I need to know I’m going to have [methadone] and that I’m going to have good medical treatment”. (Enrique, Oaxaca)*

The lack of public care in people’s communities of origin, the lack of medication (because of restrictions or lack of availability), the lack of competent medical care in managing methadone, and the lack of safety where health care is provided and received, and, in sum, the lack of a treatment program for heroin users represents a failure to guarantee their right to health care. This negligence and denial of health care leads to relapses. Such denial for structural reasons of access to treatment with proven biomedical benefits has been called “structural violence in health” [45].

## Discussion

Heroin users must undertake a pilgrimage in their search for help, and this delays their recovery process. The pilgrimage is made necessary by conditions like the stigma surrounding substance use, the existence of differing ideas about treatment, the lack of regulation for addiction care services, and the lack of access to comprehensive evidence-based treatment.

Heroin users are forced to leave their communities by organized crime and violence and the lack of specialized public clinics that provide methadone treatment, including in prison. Moradi [46] has reported benefits from providing such treatment in prison, including reduced access to drugs, including those brought in by prison personnel, and reductions in secondary effects, prisoners’ expenditure on drugs, access to injectable drugs, and reentry to prison.

The inclusion of a gender perspective is essential to encourage women’s access to drug treatment. This perspective has been neglected because of the low visibility of women’s needs and trajectories and the comorbidity with the emotional consequences of violence. Female opiate users represent a challenge for the medical profession, particularly during pregnancy and breastfeeding [47, 48]. The handling of methadone during pregnancy is a controversial issue which has received little attention in Mexico. However, in other countries, methadone maintenance treatment has been found to provide a constant concentration of opiates in the blood of pregnant women, preventing the damaging effects on the fetus of repeated withdrawal [49].

The experience of living in dangerous environments, including the constant mistreatment experienced in prison, combined with the easy availability of heroin and the lack of methadone treatment which may exacerbate the problems of substance use, calls for different intervention and policy strategies where harm reduction, however necessary, may be insufficient [19, 50].

Health care personnel need to be trained to detect, treat, and refer users with opiate dependence [39, 44]. Alternative substitution and emergency treatment, such as with buprenorphine or naloxone, is also needed, as it allows users to choose the treatment which best suits their everyday lives, as well as reducing the associated costs, such as transportation and the loss of income from taking time off to visit clinics [40].

A potential solution, derived from this analysis, is a compassionate use strategy that allows for heroin users to receive methadone treatment. Compassionate use implies the use of experimental medications in certain patients, linked to clinical trials, including prescriptions for indications or conditions other than those specifically authorized, when physicians consider them indispensable. It is used where there are no effective treatments for certain illnesses, or where there is a lack of interest or difficulty in conducting clinical trials for uncommon illnesses or with some population groups, such as children or pregnant women [51, 52]. For these reasons, there are only a few clinical trials in Mexico that have shown methadone to be a beneficial treatment for heroin addiction. However, methadone does meet the requirements for compassionate use, as a temporary strategy to provide necessary and urgent care for this group of substance users.

There is no compassionate use program or equivalent in current Mexican law, but there are two norms, RD 223/2004 [53], which regulate clinical trials of pharmaceuticals (Arts. 28 & 29, Decreto 29ii, 2006), and Decreto 29/2006 [54] regulates the use of medication and health products (Art. 24). Although this resource could benefit this group of substance users, it is not employed because health care providers are unaware of the available legal procedure, because they are unwilling to do the paperwork, because the current regulations are unclear, or because they do not recognize opiate dependence disorder as a chronic condition requiring the incorporation of methadone treatment into health care systems [44].

The presence of the researcher in the clinical environment and its methodological implications are important to mention. The view of the observer, participant or not, goes in two directions; it is a mutual questioning of the ethnographer and their informants, given that it is carried out through encounters, interactions, and conversations. Thus, reflexivity, also called biographical reflexivity, is defined not only by ascriptive characteristics, but also by the perceptions and judgments of others concerning the researcher. The people themselves who are actively involved give meaning to their own actions, which includes the research process itself: the mutual understanding between researchers and the actors who are being investigated [55].

Reflexivity can be defined in this way, as a consciousness of the mutual relationship between researcher and the object of study that shows how new ideas are generated, how the relationship is shaped by preexisting knowledge, and how research results are obtained [55]. The identity of the researcher has implications at all levels, including those of gender, ethnicity, and status that are involved in the process of research. There are also interactions that provoke feelings and emotions during field work, which provide a look at the social dynamics of a culture, a group, or the life of a subject [56]. In this process there are therefore three levels of subjectivity and thus of reflexivity: that of the subject of the experience, that of the subject that knows, the epistemic subject, and that of language in a close relationship between the epistemic subject and the subject of the experience [57].

For the first author, the field work posed several challenges, among them working in an environment assumed to be dangerous (because of the murder of the physician and threats received in other clinic locations), with a vulnerable population (and at the same time with a certain capacity for agency for what they had to do to obtain treatment; not all substance users are willing to embark upon that journey) and working alone. Her understanding of the research process came from different approaches and disciplines, such as clinical psychology, anthropology, research on gender and health services, and qualitative research. Her training as a clinical psychologist, which helped with control in emotionally difficult situations, and 30 years of experience in research on substance abuse served as useful tools. However, working in direct contact with people from different socioeconomic and cultural contexts, and conversing for the first time with heroin users required questioning and rethinking certain prejudices.

The strategy of immersion in the field was to be constantly present, noted both by clinic staff and by substance users, in a non-place, neither here nor there. The first interaction took place in the street outside the clinic. Finding it closed, I sat on the sidewalk waiting for it to open, and about six substance users arrived, one with his wife, one after another, and sat next to me, like birds on a wire. The first question they asked me was: “And you, are you also here for your dose?” This made me certain that dialogue would be possible.

There was mutual curiosity and surprise as well, with frequent contrasts between substance users who were poor, malnourished, without having eaten for days, Mexicans and foreigners, in front to those who arrived in their own cars with company. Astonishment among the substance users themselves: “If they have everything, why do they need methadone?” (Vittor, Oaxaca).

The majority liked the methodology of being interviewed: as subjects of experience—from sometimes living without being seen or heard, excluded, monitored, or arrested—to being recognized:

*“Thank you for at least being interested in learning a little about my life, among thousands. You’re the only person who has asked me, except when I’ve been arrested. Because unfortunately, the rest don’t care about what we live, what we feel, what we experience, until we are no longer...until we’re a target, unfortunately. And what you are doing is very valuable, because at least it tells a little about the reality we live. And not theoretically speaking, but from real-life experience”. (Héctor, Oaxaca)*

In the process of analysis, readings by other researchers made it possible to reflect on ideas that would have escaped the notice of a single person. Almanza, a collaborator on this study, shared his experience:

*“I did not have a direct encounter with the substance users: I was only a witness, the most empathetic one I could be, based on what I read, what you told me, and the way in which you told me. I live in another part of the country, up to now I have not used heroin and I have not been in a process of rehabilitation. I suppose I am in a position of greater privilege in terms of education and maybe of socioeconomic level and ethnicity. Although I have a clinical view, in looking at their interviews, I was interested in de-pathologizing their experience and focusing on it as a form of social suffering, as Das and Kleinman propose. As a user of health services, and a witness of their limitations, I was especially sensitive to identifying the barriers that appeared in their interactions with them. My prior experience with persons with HIV has sensitized me to the implications of stigma in health care and the way in which these services require a person to become an autonomous, independent patient, although there are structural conditions that limit their staying in treatment. And finally, having lived in places where organized crime influences urban dynamics, and I see every day now how people use drugs in the street, I sought to focus on the way in which the state has abandoned substance users, in complicity with drug trafficking. I suppose that from my previous journeys, my education, and my current everyday experience, ideas emerge that I seek to identify in the stories I read and listen to”.*

Dialogue with the women, especially the younger ones, was difficult, involving multiple attempts and finally acceptance. Our age difference was probably one factor in play; competition and/or rivalry between women, a recurrent gender theme, might have been another. Perhaps if they had been interviewed by a man, or could have chosen who would interview them, the process would have been smoother.

The vulnerability of the researcher in the field was less in the dangerous situation than in the stories in which violence was ubiquitous, as well as in the observation of bodies emaciated and injured by multiple injections, the lost gazes, and the reminders of death, which made its appearance on four occasions. Over time, parents, partners, and clinic staff all asked to be interviewed. These requests made it clear that it is not safe or easy to live around heroin: not for its users, not for health care providers, not for researchers.

## Conclusions

Current intervention strategies may overflow prisons, enforce repressive practices against substance users, contribute to discriminatory attitudes, marginalize poor people, and cause enormous suffering by failing to treat physical and emotional pain [58]. Methadone use, however, could bring about benefits that exceed the risks, although this evaluation must be made given the course and prognosis of the illness, as well as the impact on other areas of substance users’ health care. Large-scale compassionate use of methadone should follow scientific and bioethical criteria. However, sociopolitical forces, such as the influence of drug trafficking organizations, could thwart these efforts [59]. Prison sentences could provide an opportunity for methadone treatment, reducing its use or discontinuing it, and help substance users reenter society. Given the current impossibility of providing such treatment at public clinics, subsidized private treatment could afford the possibility of better treatment adherence and harm reduction. Finally, both the gender and human rights perspectives are essential to policy-making.

## Supplementary Information

**Additional file 1.** Full Interview Guide.

## Data Availability

The interviews for this study are not publicly available for reasons of privacy, confidentiality, and anonymity of the participants.

## Abbreviations

COFEPRIS: Comisión Federal para la Protección contra Riesgos Sanitarios (Federal Commission for Protection Against Health Hazards)
HIV: Human immunodeficiency virus
