# Supplementary material for: Structural violence and the need for compassionate use of methadone in Mexico
Source: BMC Public Health. 2022 Mar 29;22:606. doi: 10.1186/s12889-022-12955-x (PMC8962103; doi:10.1186/s12889-022-12955-x)
Supplement: Supplementary file 1 — Additional file 1. Full Interview Guide. [file 12889_2022_12955_MOESM1_ESM.docx]

**Full Interview Guide**

|  |
| --- |
| 1. How did you start using heroin?   Age at which you started using any drugs  Age at which you started using heroin  Type of heroin used (color, form, odor)  Methods of use (and paraphernalia used)  Frequency of use  Needle sharing  Problems caused by use (physical, social, family, legal, emotional)  Reasons for use  Cost  Pleasant effects  Unpleasant effects  Social context of use  HIV seropositivity   1. How did you start going to the methadone clinic?   What was going on in your life that made you decide to seek help?  Where did you seek help before coming to the clinic?  How did you find out about the clinic?  How did you find out about methadone?  What effects do you experience from methadone?  What are the problems you have in using methadone?  Costs  Availability |
